# *Insm1 *promotes the transition of olfactory progenitors from apical and proliferative to basal, terminally dividing and neuronogenic

**DOI:** 10.1186/1749-8104-6-6

**Published:** 2011-02-01

**Authors:** Jason N Rosenbaum, Anne Duggan, Jaime García-Añoveros

**Affiliations:** 1 Department of Anesthesiology, Northwestern University Feinberg School of Medicine, 303 E. Chicago Avenue, Ward 10-070, Chicago, IL 60611, USA; 2 Department of Neurology and Physiology and The Hugh Knowles Center for Clinical and Basic Science in Hearing and its Disorders, Northwestern University Feinberg School of Medicine, 303 E. Chicago Avenue, Ward 10-070, Chicago, IL 60611, USA

## Abstract

**Background:**

Insm1 is a zinc-finger transcription factor transiently expressed throughout the developing nervous system in late progenitors and nascent neurons. Insm1 is also highly expressed in medulloblastomas and other neuroendocrine tumors.

**Results:**

We generated mice lacking the *Insm1 *gene and used them to elucidate its role in neurogenic proliferation of the embryonic olfactory epithelium. We found that deletion of *Insm1 *results in more apical cells and fewer nascent and mature neurons. In the embryonic olfactory epithelium of *Insm1 *mutants we detect fewer basal progenitors, which produce neurons, and more apical progenitors, which at this stage produce additional progenitors. Furthermore, in the mutants we detect fewer progenitors expressing NEUROD1, a marker of terminally dividing, neuronogenic (neuron-producing) progenitors (immediate neuronal precursors), and more progenitors expressing ASCL1, a marker of the transit amplifying progenitors that migrate from the apical to the basal edges of the epithelium while dividing to generate the terminal, neuronogenic progenitors. Finally, with timed administration of nucleoside analogs we demonstrate that the *Insm1 *mutants contain fewer terminally dividing progenitors at embryonic day 12.5.

**Conclusions:**

Altogether, these results suggest a role for *Insm1 *in promoting the transition of progenitors from apical and proliferative to basal, terminal and neuronogenic. This role appears partially conserved with that of its nematode ortholog, *egl-46*. The similar effects of *Insm1 *deletion on progenitors of embryonic olfactory epithelium and cortex point to striking parallels in the development of these neuroepithelia, and particularly between the basal progenitors of olfactory epithelium and the subventricular zone progenitors of cortex.

## Background

During nervous system development, progenitors divide to produce more progenitors, glia and/or neurons. Decisions to stop proliferating and produce neurons or glia determine shape, size and cellular composition of every part of the nervous system. These decisions can involve transitions between apical and basal sides of a proliferating neuroepithelium. In embryonic olfactory epithelium (OE), apical progenitors generate sustentacular glia and more progenitors, some of which transition basally, divide terminally and produce neurons [1]. Despite its importance, we have limited knowledge of which genes control progenitor transitions from proliferative to neuronogenic.

In *Caeneorhabditis elegans*, the zinc-finger protein EGL-46 is transiently expressed in certain progenitors about to divide terminally (neuronogenic, N/N divisions) and in their two nascent neurons, but not in the mature neurons or in earlier progenitors that divide to produce one or two progenitors (proliferative, P/P or P/N divisions) (see discussion below). In *egl-46 *mutants, some of these presumptive N/N progenitors fail to terminally divide and generate additional progenitors [2-4] (see discussion below). Hence, EGL-46 regulates terminal neuronogenic divisions. We hypothesize that a mammalian homolog of EGL-46 also regulates transitions of progenitors from proliferative to terminally dividing and neuronogenic.

In mammals, *egl-46 *has two orthologs, *Insm1 *and *Insm2 *[3,5]. *Insm1 *was originally identified as highly expressed in neuroendocrine tumors [6-13], but during normal development it is transiently expressed throughout the embryonic and adult developing nervous system [14-18]. In particular, *Insm1 *mRNA is expressed in late (not early) progenitors and nascent (not mature) neurons [15]. Thus, the pattern of expression of *Insm1 *in mice is reminiscent of that of *egl-46 *in nematode neuronal lineages, suggesting that both genes share a conserved function in regulating neuronal progenitor proliferation. *Insm1 *has been implicated in the development of the pancreas [19-22], sympatho-adrenal lineages [23], cortex [16], and hindbrain [24]. We tested the function of this gene in the embryonic OE.

The OE provides a simple neurodevelopmental model. This placode-derived neuroepithelium contains only one type of neuron, which originates from progenitors located within the epithelium. Embryonic OE is spatially segregated: early progenitors and sustentacular cells localize apically, late progenitors basally, and neurons in between [1,25]. In embryonic OE, *Insm1 *mRNA is expressed in intermediate cells (both progenitors and nascent neurons) and basal (but not apical) progenitors [15]. We generated mice entirely lacking *Insm1 *and used them to conclude that this gene regulates progenitor transitions from apical and proliferative to basal and neuronogenic.

## Results

**Figure 1:**
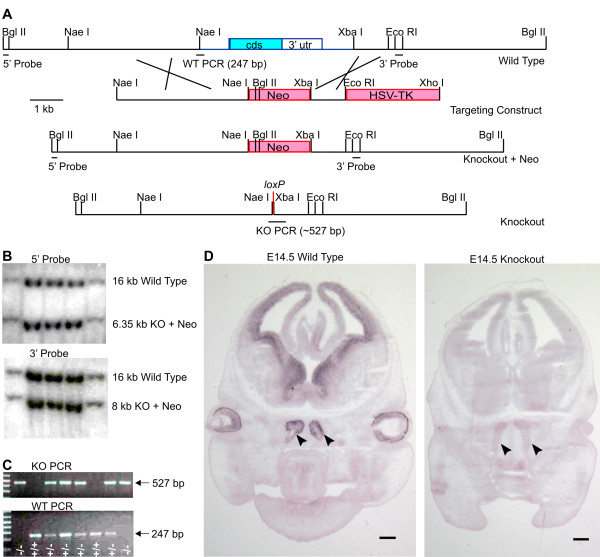
**Generation of *Insm1 *knockout mice**. **(A) **Schematic of the recombination strategy used to generate the *Insm1 *knockout. Using homologous recombination in embryonic stem (ES) cells, we replaced a genomic DNA fragment of 3.4 kb (in blue/cyan) containing the entire single exon of *Insm1 *plus 927 bp of upstream and 955 bp of downstream sequence with a 'floxed' neomycin cassette (in red/pink). After generating chimeric mice and, from these, heterozygotes, the neomycin cassette was removed by crossing with *E2a-Cre *transgenic mice. Probes (5' and 3') used for Southern blot genotyping and DNA fragments obtained by PCR genotyping of the wild type, knockout + neomycin and knockout alleles are depicted. **(B) **Southern blots of *Bgl*II digests of genomic DNA from recombined ES cells with 5' and 3' probes reveal the expected bands from the knockout (KO) and wild type alleles and confirm that recombination was complete. **(C) **Genotyping PCR using genomic DNA obtained from a litter of embryos (embryonic day 10.5) resulting from crossing two heterozygous parents. Primers used are described in methods and their products depicted in (A). The litter contained two knockouts (-/-), two wild types (+/+) and four heterozygotes (+/-), as indicated in the figure. **(D) ***In situ *hybridization (ISH) with an antisense probe against the 3' UTR of *Insm1 *on wild type and knockout littermate embryos (E14.5, coronal sections) reveal the presence of *Insm1 *mRNA in the brain, retina and olfactory epithelium (arrowheads) of wild type but not of knockout embryos, functionally confirming the knockout of *Insm1*. Scale bars: 500 μm.

In order to determine the role of *Insm1 *in neuronal development, we generated knockout (KO) mice lacking this gene. The *Insm1 *mRNA is transcribed from a single exon (2,912 bp long) containing 155 bp of 5' UTR, 1,566 bp of coding sequence and 1,206 bp of 3' UTR. By homologous recombination in HM1-M embryonic stem (ES) cells (derived from Sv129 mice), we replaced 3.4 kb of genomic DNA containing the entire *Insm1 *exon with a neomycin cassette surrounded by *loxP *sites for future excision (Figure 1A). Several of these recombined ES cell clones were injected into C57BL/6 blastocysts and multiple chimeras were generated. Two (out of 17) chimeras generated from one of these ES cell clones (11G9) passed the *Insm1*^*tm1Jga *^allele (which bears a 'floxed' neomycin cassette in place of *Insm1*) through the germ line and produced heterozygous progeny. We generated the final KO allele, *Insm1*^*tm1.1Jga*^, by crossing these heterozygotes with an E2a-Cre transgenic mouse, which express the Cre recombinase ubiquitously, thus removing the neomycin cassette and leaving only a *loxP *site in place of the wild type allele of *Insm1 *(Figure 1A). We confirmed deletion of *Insm1 *by Southern blot and by PCR (Figure 1B, C). Furthermore, *in situ *hybridization revealed that the mRNA of *Insm1*, detected in wild type embryos in the expected neuronogenic areas (including OE) [15], was missing in KO embryos (Figure 1D). These results, in addition to confirming the functional KO of the *Insm1 *gene, also demonstrate that the distribution reported with this same probe, in late progenitors and nascent neurons ([15] and data not shown), does indeed represent the mRNA of *Insm1*, not of a homolog.

To avoid potentially confounding effects of other genetic variables in our phenotypic assessment, we placed the *Insm1*^*tm1.1Jga *^allele (from now on referred to as *Insm1*^-^) into an isogenic background by performing ten backcrosses into either the inbred C57BL/6 line or the outbred CD1 line.

### Deletion of *Insm1 *causes embryonic lethality

Mating between heterozygotes did not result in any *Insm1*^*-/- *^pups, which indicates that this genotype causes embryonic lethality. This lethality does not seem to result in or from developmental delay, as KO, wild type and heterozygous embryos of the same age did not differ in weight (Additional file 1) or in anatomically defined developmental hallmarks (Additional file 2). In order to determine the embryonic stage of lethality, we generated timed pregnancies and collected embryos at set days post-coitus (from here onwards denoted as E for embryonic day, with the first day after a night of mating considered E0.5). At E11.5, *Insm1*^*-/- *^embryos appeared at the expected Mendelian ratio (1/4). At E12.5 to E15.5, *Insm1*^*-/- *^embryos appeared at somewhat lower ratios than expected and by E16.5 there was a severe drop in viability (Additional file 3). Hence, *Insm1*^*-/- *^embryos suffer lethality around E16. In an attempt to obtain older embryos, we administered L-DOPA (1 mg/ml) to pregnant dams starting no later than 7.5 days post-coitus (dpc), as it has been reported that this treatment increases viability of a similar KO [23,26]. Although in our hands this treatment did not improve the viability at earlier (E11.5 to E14.5) stages, we were able to obtain a few embryos as old as E20.5 (Additional file 3). We used these embryos to examine the effects of *Insm1 *deletion on neural development but we did not explore the reasons for this lethality, which others attribute to a catecholamine deficiency resulting from developmental defects of the sympathoadrenal lineage [23].

### Deletion of *Insm1 *results in more apical and fewer neuro-basal cells in the embryonic OE

**Figure 2:**
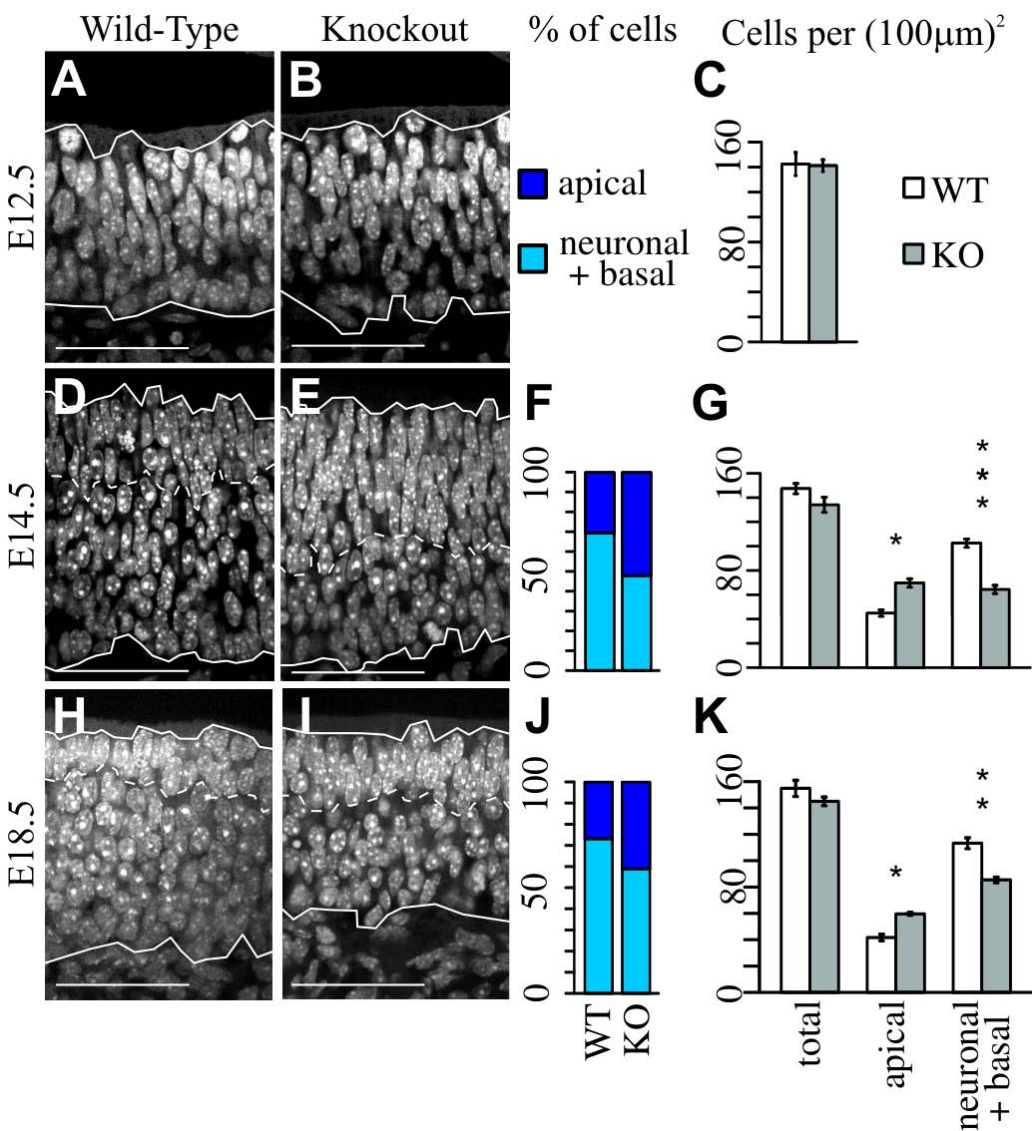
**Increase in the number of apical cells and concomitant decrease in the number of neuro-basal cells in the OE of *Insm1*^*-/- *^embryos**. Representative DAPI stained images from coronal sections of **(A, D, H) ***Insm1*^*+/+ *^embryos and **(B, E, I) **anatomically aligned sections from paired *Insm1*^*-/- *^littermates. Solid lines indicate the apical (top) and basal (bottom) margins of the OE, and dashed lines indicate the interior border of the apical compartment. In *Insm1*^*-/- *^mice at (**D-G**) E14.5 and (**H-K**) E18.5, the number of apical cells per (100 μm)^2 ^of total OE surface is 57% greater (n = 4 embryo pairs, total of 12 aligned section pairs, *P *< 0.05) and 44% greater (n = 3 embryo pairs, total of 9 aligned section pairs, *P *< 0.05), respectively, than in paired *Insm1*^*+/+ *^littermates. Correspondingly, the number of neuro-basal cells decreases by 37% at E14.5 (*P *< 0.001) and by 24% at E18.5 (*P *< 0.01). As a percentage of total cells, at E14.5 apical cells make up 30% of the OE in *Insm1*^*+/+ *^embryos and 52% in *Insm1*^*-/- *^embryos (F). At E18.5, apical cells make up 27% in *Insm1*^*+/+ *^embryos and 41% in *Insm1*^*-/- *^embryos (J). At E12.5, the apical and basal layers are not yet apparent. However, there is no detectable difference between the overall thickness of the OE at any stage (*P *= 0.98 at E12.5, *P *= 0.07 at E14.5 and *P *= 0.17 at E18.5; n = 3 embryo pairs, total of 9 aligned section pairs at E12.5). Data in (C, G, K) are presented as mean values ± standard error of the mean (SEM). *P ≤ 0.05, **P ≤ 0.01, ***P ≤ 0.001. Scale bars: 50 μm. KO, knockout; WT, wild type.

After close examination of paired sections of KO and wild-type littermate embryos, we focused our attention on the OE, which appeared altered in the KO. The embryonic OE at E14.5 and later stages contains two cellular layers that are easily distinguished based on nuclear morphology: apical and neuro-basal (Figure 2D, H). The apical layer consists of densely packed, elongated nuclei that correspond to proliferative progenitors. These apical progenitors undergo mitosis at the luminal side of the epithelium and initially generate additional progenitors, some of which migrate basally to divide terminally and produce neurons [1,25,27]. Eventually, progenitors that remain in the apical layer generate the apically located sustentacular (glial-like) support cells [28]. The neuro-basal layer consists of rounder, more densely spaced nuclei that correspond to basal progenitors and to their progeny, the intermediately located neurons. With a nuclear DAPI stain we detected no change in the thickness of the entire OE at E12.5, E14.5 and E18.5 (Additional file 4). However, the apical layer of proliferative progenitors was thicker in *Insm1*^*-/- *^than in *Insm1*^*+/+ *^embryos at E14.5 (+47%) and E18.5 (+59%), and the neuro-basal layer was conversely thinner (Additional file 4). Nuclear counts confirm that, compared to *Insm1*^*+/+ *^littermate OEs, *Insm1*^*-/- *^OEs contained more apical cells (+57% at E14.5 and +44% at E18.5, measured per unit area of the whole OE) and fewer neuro-basal ones (-37% at E14.5 and -24% at E18.5, measured per unit area of the whole OE) (Figure 2).

### Deletion of *Insm1 *results in fewer nascent and mature neurons in the embryonic OE

**Figure 3:**
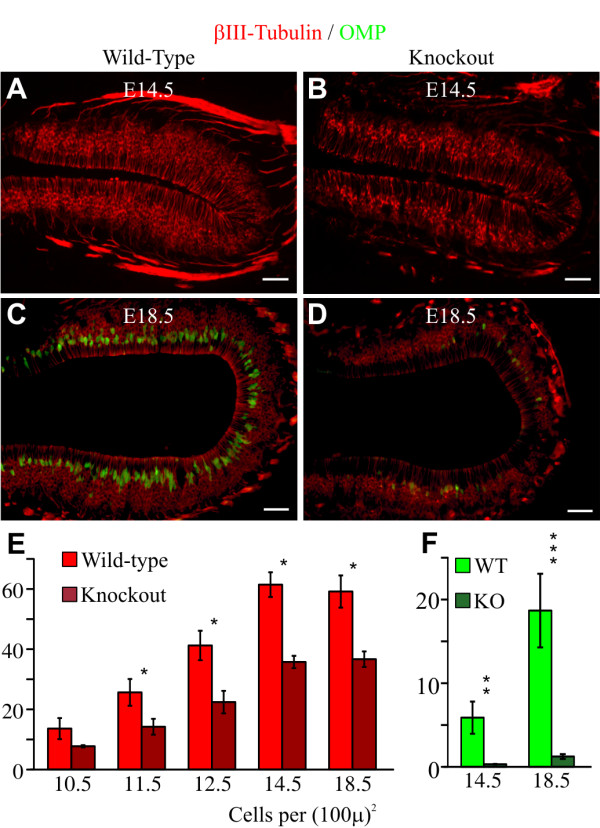
**Fewer cells express markers of young (βIII-tubulin) and mature (olfactory marker protein) olfactory receptor neurons in the OE of *Insm1*^*-/- *^embryos**. **(A-D) **Immunohistochemistry for βIII-tubulin (red) plus **(C-D) **olfactory marker protein (OMP; green) in representative, anatomically aligned images from the OE of (A, B) E14.5 and (C, D) E18.5 *Insm1*^*-/- *^and an *Insm1*^*+/+ *^littermate embryo. Fewer cells express either marker in the knockout (KO). The apical border is toward the center of each panel, and the basal lamina is toward the periphery. **(E) **Density of young neurons (number of cells expressing βIII-tubulin per (100 μm)^2^) in OE at various embryonic stages. In *Insm1*^*-/- *^mice at E11.5, E12.5, E14.5, and E18.5 the number of young neurons (red) is reduced by an average of 43% (n = 3 embryo pairs, total of 9 aligned section pairs, *P *< 0.05), 46% (n = 3 embryo pairs, total of 9 aligned section pairs, *P *< 0.05), 39% (n = 4 embryo pairs, total of 15 aligned section pairs, *P *< 0.01), and 37% (n = 3 embryo pairs, total of 9 aligned section pairs, *P *< 0.05), respectively. At E10.5, the measured reduction (29%, n = 3 embryo pairs, total of 9 aligned section pairs) does not achieve statistical significance. **(F) **Density of mature neurons (number of cells expressing OMP per (100 μm)^2^) in OE at various embryonic stages. At E14.5 and E18.5 the number of mature olfactory neurons is reduced by 92% (n = 3 embryo pairs, total of 9 aligned section pairs, *P *< 0.01) and 94% (n = 3 embryo pairs, total of 9 aligned section pairs, *P *< 0.001), respectively. Data in (E, F) are presented as mean values ± standard error of the mean (SEM). *P ≤ 0.05, **P ≤ 0.01, ***P ≤ 0.001. Scale bars: 50 μm. WT, wild type.

The reduction in neuro-basal cells observed in the OE of *Insm1*^*-/- *^embryos could reflect a reduction in basal progenitors, in neurons, or in both. We labeled nascent neurons with antibodies against the βIII-tubulin isoform (TuJ1). At most stages examined, the *Insm1*^*-/- *^OEs had significantly fewer nascent (TuJ1+) neurons than the OEs of their *Insm1*^*+/+ *^and *Insm1*^*+/- *^littermates (-43% at E11.5, -47% at E12.5, -39% at E14.5, and -37% at E18.5; Figure 3). At E10.5, the small reduction measured is not statistically significant. Hence, we conclude that as early as E11.5 there are fewer neurons in the OEs of *Insm1*^*-/- *^embryos than of *Insm1*^*+/+ *^and *Insm1*^*+/- *^littermates. We also labeled with antibodies to the olfactory marker protein (OMP), a marker of mature olfactory receptor neurons, which are detected as early as about E14 [29-31]. We also found a reduction (-92 to -94%) of OMP+ cells at E14.5 and E18.5 (at earlier stages there is little or no OMP expression) (Figure 3). Hence, deletion of *Insm1 *results in fewer nascent and mature neurons in the embryonic OE.

### Deletion of *Insm1 *only causes a minimal increase in apoptosis at late embryonic stages

**Figure 4:**
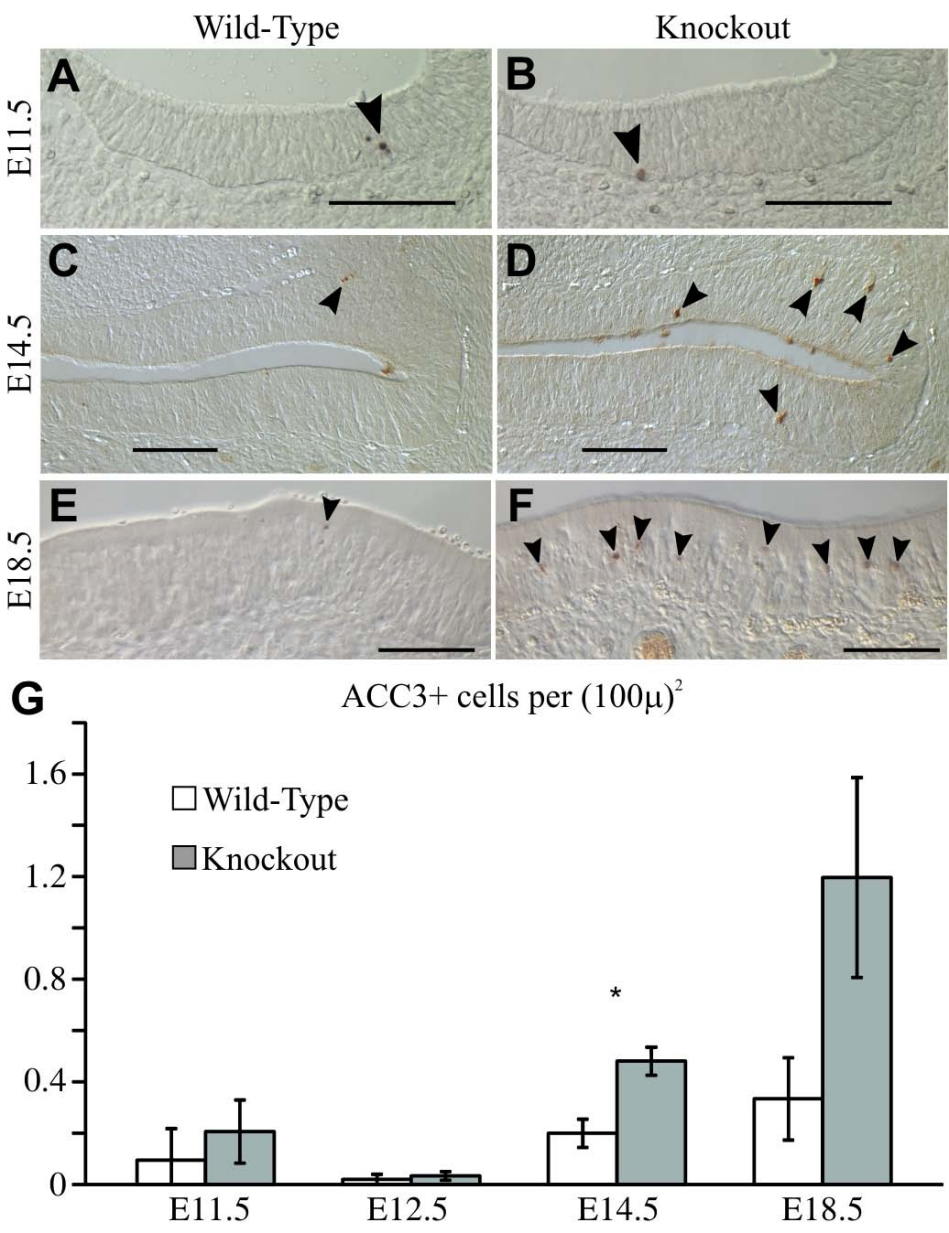
**Increased apoptosis in the OE of *Insm1*^*-/- *^embryos does not precede the decrease in nascent neurons**. **(A-F) **Immunohistochemistry for activated cleaved caspase 3 (ACC3), a marker of apoptotic cells, at selected stages. ACC3+ staining is indicated by arrowheads. **(G) **At E11.5 (and compare (A) and (B)) and E12.5, there is no significant difference in ACC3+ cells (n = 3 embryo pairs, *P *= 0.63). By E14.5 (compare (C) and (D)), more ACC3+ cells are detected in *Insm1*^*-/- *^mice than in their paired *Insm1*^*+/+ *^littermates (n = 3 embryo pairs, *P *< 0.05), an increase that persists at E18.5 (compare (E) and (F); n = 3 embryo pairs, *P *= 0.11). Data in (G) are presented as mean values ± standard error of the mean (SEM). *P ≤ 0.05. Scale bars: 100 μm.

A decrease in neurons could be caused by an increase in cell death. In the embryonic OE of the mouse, there are two waves of apoptosis. The first peaks at E11.5 and is thought to correspond to morphogenetic changes. The second ranges from E13.5 to E17.5 (peaking at E15.5) and corresponds to innervations of olfactory receptor neurons with their targets in the olfactory bulb. Both nascent and mature olfactory receptor neurons undergo apoptosis during development [32]. We labeled apoptotic cells with antibodies against activated cleaved caspase 3 (ACC3) and confirmed the low levels of apoptosis at E12.5 and the higher levels at E11.5 and E14.5 (Figure 4). Compared to *Insm1*^*+/+ *^littermates, *Insm1*^*-/- *^embryos displayed increased apoptosis in their OEs at E14.5 (*P *< 0.05) and perhaps also at E18.5 (*P *= 0.11), but not at E12.5 (*P *= 0.62) or E11.5 (*P *= 0.6). Therefore, the reduction of neurons (detected at E11.5) precedes by at least 2 days any increase in apoptosis. Furthermore, the number of additional apoptotic cells at E14.5 in the *Insm1*^*-/- *^OE is approximately 100 times lower than the number of missing neurons (compare Figures 4G and 3E). Hence, although apoptosis might contribute slightly to the reduction of neurons, it is not the primary cause of this reduction.

### Deletion of *Insm1 *does not alter cell cycle length, but results in an increase in apical progenitors and a concomitant decrease in basal ones in the embryonic OE

**Figure 5:**
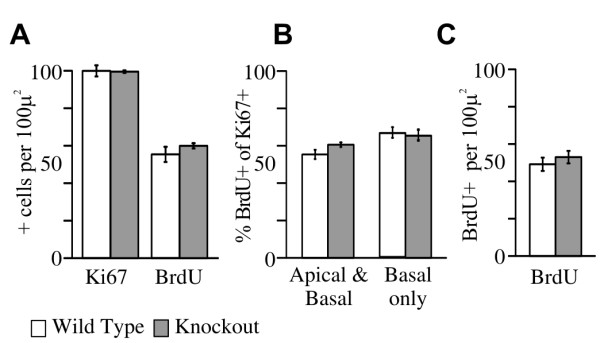
**The number of OE progenitors in the cell cycle or S-phase, as well as cell cycle length, is unaltered in *Insm1*^*-/- *^embryos at E12.5**. **(A) **At E12.5 there is no difference in the number of OE cells in the cell cycle (those expressing Ki67; *P *= 0.91) or in S-phase (those having incorporated bromodeoxyuridine (BrdU) 30 minutes after injection; *P *= 0.29) between *Insm1*^*-/- *^(grey bars) and *Insm1*^*+/+ *^littermates (white bars; n = 3 embryo pairs, total of 9 aligned section pairs). **(B) **The fraction of dividing cells (Ki67+) that are in S-phase (BrdU+), which is indicative of cell cycle length, does not differ between *Insm1*^*-/- *^mice and their *Insm1*^*+/+ *^littermates (*P *= 0.11). Cell cycle length does not differ among genotypes when considering all progenitors (apical + basal; *P *= 0.11) or only the basal ones (*P *= 0.19). **(C) **For BrdU incorporation, a larger data set (n = 6 embryo pairs, total of 18 aligned section pairs) also shows no significant difference of cells in S-phase at E12.5 (*P *= 0.08). Data are presented as mean values ± standard error of the mean (SEM).

A reduction in neurons might also result from a change in progenitor proliferation. A decrease in progenitors or an increase in their cell cycle length would result in a decrease in nascent neurons. Paradoxically, an increase in progenitors might also result in a temporary reduction of neurons produced if, as in the *egl-46 *mutant nematodes, progenitors continue dividing instead of producing two neurons [2-4]. We labeled OE progenitors at E12.5 with Ki67 antibodies (which marks cells at all phases of the cell cycle) [33-35] and by 30 minutes of 5-bromo-2-deoxyuridine (BrdU) incorporation (which labels all cells in S-phase) and found no difference in the overall number of progenitors between *Insm1*^*-/- *^and *Insm1*^*+/+ *^littermates (Figure 5).

Because the length of the S-phase is largely invariant and changes in cell cycle length reflect changes in G1, we could estimate changes in overall cell cycle length by calculating the fraction of all dividing cells (Ki67+) that are in S-phase (BrdU+) [36]. A lengthening of the cell cycle, which would result in a delay in the production of neurons, would be reflected in a lower BrdU+/Ki67+ ratio. However, we detected no difference in this ratio when comparing OEs of *Insm1*^*-/- *^and of *Insm1*^*+/+ *^littermate embryos (Figure 5). Hence, we conclude that the deletion of *Insm1 *does not cause an overall lengthening of the cell cycle among OE progenitors.

The above measurements did not distinguish between apical and basal progenitors, and would not have detected a cell cycle lengthening in basal progenitors (which could explain the reduction in neurons) if this was compensated by a cell cycle shortening in apical progenitors. However, we also detected no difference in BrdU+/Ki67+ ratios when considering only basal progenitors (Figure 5). We therefore conclude that the reduction of olfactory receptor neurons in *Insm1*^*-/- *^embryos is not caused by a lengthening of the cell cycle in basal OE progenitors.

**Figure 6:**
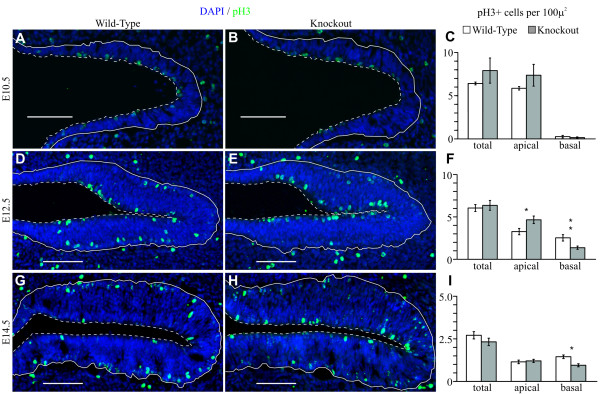
**Mitotically active (pH3+) progenitors decrease basally and increase apically in the OE of *Insm1*^*-/- *^embryos**. **(A**,**D**,**G) **Representative images from coronal sections of *Insm1*^*+/+ *^mice. **(B**,**E**,**H) **Anatomically aligned section pairs from paired *Insm1*^*-/- *^littermates. Dorsal is toward the right, ventral toward the left. **(A-C) **At E10.5, there is no statistically significant difference in pH3+ cells overall, or at either the apical or basal border of the OE (n = 4 embryo pairs, total of 12 aligned section pairs, *P *= 0.36 overall, *P *= 0.25 apically, *P *= 0.57 basally). **(D-F) **At E12.5, *Insm1*^*-/- *^mice exhibit no difference in overall numbers of pH3+ (green) cells when compared with *Insm1*^*+/+ *^littermates, but an average 42% increase in pH3+ cells in the apical subpopulation (*P *< 0.05), and an average 45% decrease in the basal subpopulation (*P *< 0.01; n = 4 embryo pairs, total of 13 aligned section pairs). **(G-I) **By E14.5, there is still no difference in overall pH3+ cells between *Insm1*^*-/- *^mice and their paired *Insm1*^*+/+ *^or *Insm1*^*+/- *^littermates, and there is also no significant difference between pH3+ cells in the apical subpopulation. However, there remains a 35% decrease of mitotically active cells in the basal subpopulation (*P *< 0.05; n = 4 embryo pairs, total of 22 aligned section pairs). Solid white lines delineate the basal lamina and dashed lines delineate the apical edge (nasal airway). Data in (C, F, I) are presented as mean values ± standard error of the mean (SEM). *P ≤ 0.05, **P ≤ 0.01. Scale bars: 100 μm.

We also found no difference between *Insm1*^*-/- *^and *Insm1*^*+/+ *^embryos in the overall number of progenitors in mitosis, which we labeled with antibodies against phosphohistone H3 (pH3) [37] at E10.5, E12.5 and E14.5 (Figure 6). This label, however, permits us to distinguish between progenitors undergoing mitosis apically, which generate more progenitors, and progenitors undergoing mitosis basally, which after one or more divisions produce neurons [38]. In *egl-46 *mutant nematodes, there is a transient increase in proliferative (P/P) divisions at the expense of neuronogenic (N/N) divisions [2-4]. In the mouse embryonic OE, this might translate into increased apical and decreased basal progenitors. Indeed, we found an increase of apical mitoses (+42% at E12.5) and a decrease of basal mitoses (-45% at E12.5 and -35% at E14.5) in the OEs of *Insm1*^*-/- *^compared to *Insm1*^*+/+ *^littermates (Figure 6D-I). The decrease in basal mitoses could mathematically account for the decrease in neurons (Figure 3) observed in the embryonic OEs of *Insm1*^*-/- *^mice. Additionally, the increase in apical divisions may explain the increase in thickness (Additional file 4) and cells (Figure 2) of the apical layer of the *Insm1*^*-/- *^mice.

The thickening of the apical layer and thinning of the neuro-basal layer roughly compensate for each other because, as indicated above, the overall thickness of the OE does not change in *Insm1*^*-/- *^embryos. We also measured the entire surface areas of the OE sections used for estimating number of neurons (Figure 3) and confirmed no difference in the OE size of *Insm1*^*-/- *^compared to that of *Insm1*^*+/+ *^littermates (Additional file 5).

### Deletion of *Insm1 *results in more ASCL1-expressing and fewer NEUROD1-expressing progenitors in the embryonic OE

**Figure 7:**
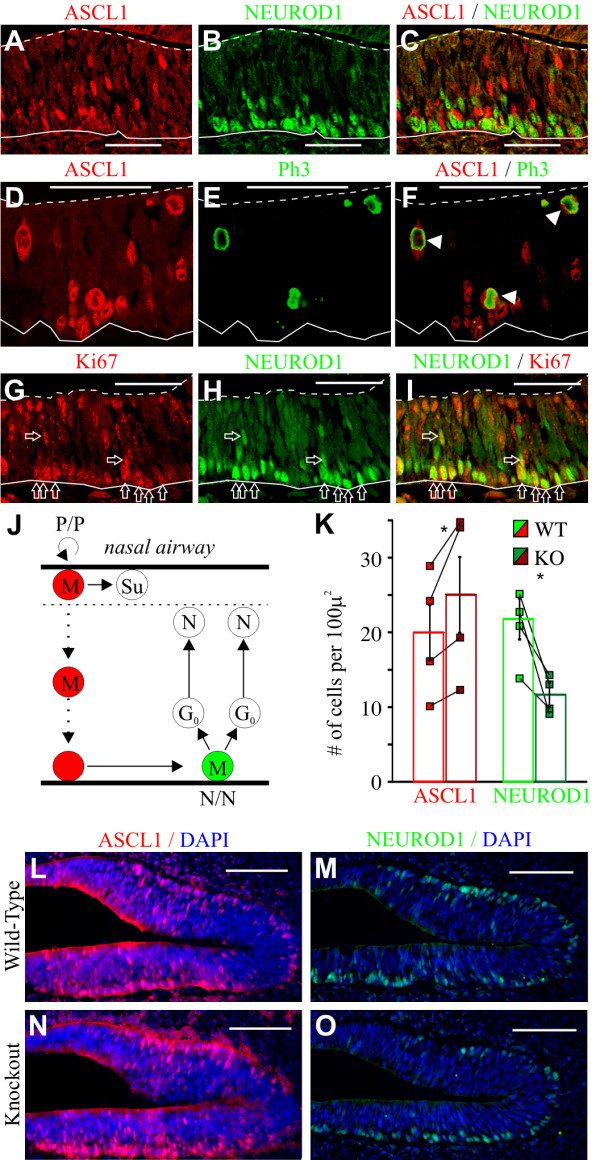
**Increase in the number of progenitors expressing ASCL1 (MASH1) and decrease in those expressing NEUROD1 in *Insm1*^*-/- *^embryonic OE**. **(A-I) **Coronal sections of E12.5 wild type OE. Solid lines delineate basal lamina and dashed lines apical edges. (A-C) Antibodies to ASCL1 and NEUROD1 label largely different subsets of progenitors. (D-F) ASCL1 is expressed in cells located across the epithelium (see also (A)), as expected for TAPs transitioning through intermediate cell layers. Some ASCL1-expressing cells at apical and intermediate positions express pH3 (arrowheads), confirming they are progenitors. (G-I) NEUROD1-expressing cells are at the basal edge and 79% of them also express the cell-cycle marker Ki67 (arrows; n = 3 sections of OE from 2 embryos), indicating that NEUROD1 is expressed in basal progenitors. **(J) **Representation of a TAP, expressing ASCL1 and migrating from the apical airways to the basal lamina, where *Ascl1 *is downregulated, *NeuroD1 *is upregulated, and NEUROD1+ cells (INPs) divide terminally to produce neurons ('N'; 'M' represents mitotic cells, G0 postmitotic cells and Su sustentacular cells). **(K, L) **Increase in ASCL1-expressing cells (*P *< 0.05; n = 4 embryo pairs, total of 12 anatomically aligned sections; compare (L) and (N)) and decrease in NEUROD1-expressing cells (*P *< 0.05; n = 4 embryo pairs, total of 12 anatomically aligned sections; compare (N) and (O)) in the OE of *Insm1*^*-/- *^embryos (compared to *Insm1*^*+/+ *^and *Insm1*^*+/- *^littermates). (K) Quantification. **(L-O) **Coronal sections of E12.5 OE from *Insm1*^*-/- *^and *Insm1*^*+/+ *^labeled with markers of nuclei (DAPI), presumed TAPs (ASCL1) and presumed terminally dividing neuronogenic progenitors (NEUROD1). Dorsal is to the right and septal toward the bottom. Data in **(K) **are presented as mean of all data (columns) ± standard error of the mean (SEM) (vertical bars). *P ≤ 0.05. Small squares depict the average density of each embryo, and values from each anatomically paired section are connected by oblique lines. Scale bars: 50 μm (A-I); 100 μm (L-O). KO, knockout; WT, wild type.

In the OE the immediate neuronal precursors (INPs), progenitors that divide terminally to produce two neurons (N/N divisions), are located basally and express the basic-helix-loop-helix transcription factor NEUROD1 [38-44]. These terminal progenitors are generated from the transit amplifying progenitors (TAPs), which in the medial and dorso-lateral OE express another basic-helix-loop-helix protein, ASCL1 (also known as MASH1) (reviewed in [1,25,41,43-45] (Figure 7J). In the embryonic OE, ASCL1-expressing TAPs appear to translocate from the apical compartment to the basal lamina and are present throughout the thickness of the OE. By immunohistochemistry (IHC) on sections of E12.5 embryos, we confirmed that antibodies raised against ASCL1 label cells located across the OE that undergo mitosis at apical and intermediate positions (Figure 7A, D-F, J). In addition, antibodies to NEUROD1 label basal cells, most of which (79%) express Ki67 and are therefore dividing (Figure 7G-J). With double IHC we confirm that ASCL1 and NEUROD1 antibodies label different populations of progenitors at this stage (Figure 7A-C). Using these antibodies, we find that in the OEs of *Insm1*^*-/- *^embryos at E12.5 (compared to *Insm1*^*+/+ *^and *Insm1*^*+/- *^littermates) there are fewer (-45%) NEUROD1-expressing cells (presumably the terminally dividing neuronogenic progenitors, or INPs) and more (+24%) ASCL1-expressing cells (presumably the TAPs transitioning from apical to basal) (Figure 7K-O).

### Deletion of *Insm1 *results in fewer terminal divisions in the embryonic (E12.5) OE

**Figure 8:**
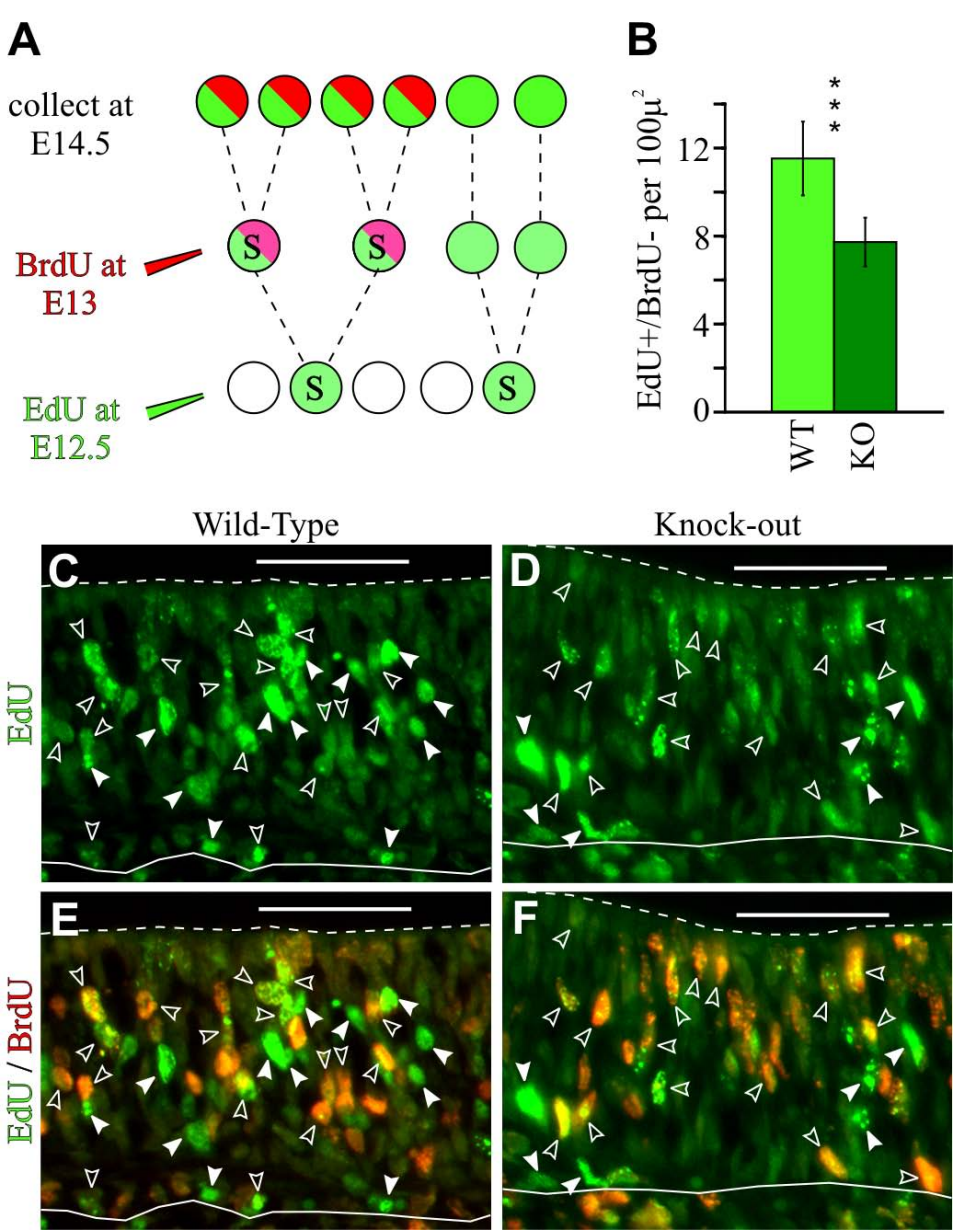
**Decrease of OE progenitors undergoing terminal division at E12.5 in *Insm1*^*-/- *^mice**. **(A) **Schematic of strategy for detecting terminally dividing cells within the embryonic OE. Pregnant dams were injected with 10 mM EdU at E12.5, followed by 10 mM BrdU 12 hours later at E13. Embryos were sacrificed at E14.5 and processed for immunohistochemical detection of BrdU and chemical detection of EdU. Cells produced by terminal division at E12.5 would be positive for EdU and negative for BrdU (green only; in the schematic as drawn, two cells out of the six). **(B) ***Insm1*^*-/- *^mice had an average of 33% fewer terminally dividing progenitors than their paired *Insm1*^*+/+ *^and *Insm1*^*+/- *^littermates (n = 3 embryo pairs, total of 9 aligned section pairs, *P *< 0.001). **(C-F) **Representative aligned coronal sections from (C, E) an *Insm1*^*+/+ *^embryo and (D, F) an *Insm1*^*-/- *^littermate. EdU label is green and BrdU label is red. Open arrowheads indicate selected double-labeled cells and solid arrowheads indicate cells labeled only with EdU (11 in wild type (WT) compared to 6 in knockout (KO)). Solid white lines indicate the basal lamina (bottom), and dashed lines indicate the apical edge (nasal airway; top). Data in (B) are presented as mean values ± standard error of the mean (SEM). ***P ≤ 0.001. Scale bars: 50 μm.

Although the reduction of NEUROD1-expressing cells is consistent with a decrease in terminally dividing (N/N) neuronogenic progenitors, this reduction could also occur if Insm1 is an activator of *NeuroD1*. In fact, Insm1 protein can bind to the *NeuroD1 *promoter, although the presumed effect is to repress it [46]. In addition, although the reduction in basal mitoses is consistent with a decrease in neuronogenic progenitors, a reduction in basal progenitors could also occur if apical progenitors fail to translocate basally but still divide terminally to produce two neurons. Hence, we sought to independently test for terminal divisions by sequential labeling of OE progenitors. We injected pregnant mothers at E12.5 with the nucleoside analog 5-ethynyl-2'-deoxyuridine (EdU) for incorporation in the DNA of cells undergoing S-phase over the following several hours. We then injected another nucleoside analog, BrdU, 12 hours later (approximately the length of one cell cycle). At E14.5, we labeled sections with OE to determine which cells that had incorporated EdU (detected by chemical fluorescence; see Materials and methods) and/or BrdU (detected by immunofluorescence) (Figure 8A). We considered that cells dividing at E12.5 that continued to divide would be double labeled (EdU+ and BrdU+), whereas progenitors terminally dividing at E12.5 would appear EdU+ but BrdU-. We found that the OEs of *Insm1*^*-/- *^embryos, compared with those of *Insm1*^*+/+ *^and *Insm1*^*+/- *^littermates, contained 35% fewer EdU+ and BrdU- cells (Figure 8). Hence, we conclude that the absence of *Insm1 *causes a decrease in terminally dividing progenitors.

### Transient expression of *Insm1 *from apical cells migrating basally to postmitotic daughters of basal progenitors

**Figure 9:**
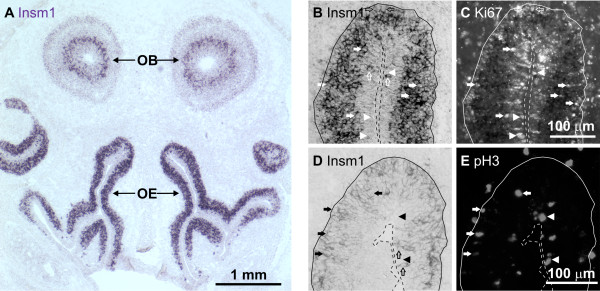
**Basal and intermediate progenitors of OE express *Insm*1 mRNA**. **(A) ***In situ *hybridization (ISH) on a coronal section of an E17.5 mouse embryo head reveals Insm1 in basal and intermediate, but not apical, cells of the OE. Subventricular cells of the olfactory bulb (OB) also express Insm1. **(B, C) **ISH for *Insm1 *(B) combined with immunohistochemistry for the cell-division marker Ki67 (C) on the same coronal sections of E14.5 OE demonstrates *Insm1 *expression in few apical (empty arrows) and most intermediate and basal cells. While most apical progenitors (Ki67-positive) do not express *Insm1 *(arrowheads), intermediate and basal cells in division do express *Insm1 *(filled arrows). **(D, E) **ISH for *Insm1 *(D) combined with immunohistochemistry for the mitotic marker pH3 (E) on the same coronal sections of E12.5 OE demonstrates *Insm1 *expression in few apical (empty arrows) and most intermediate and basal cells. While most apical progenitors in mitosis do not express *Insm1 *(arrowheads), intermediate and basal progenitors in mitosis do express *Insm1 *(filled arrows).

**Figure 10:**
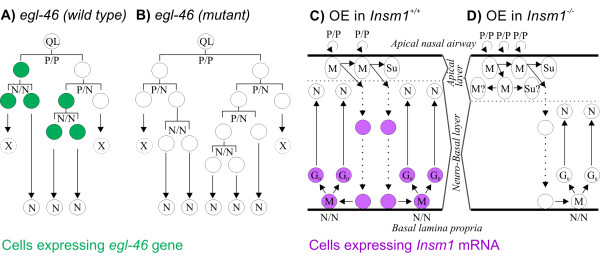
**Phenotypic similarities between *egl-46 *mutant nematodes and *Insm1 *knockout mouse OEs suggest a conserved role for both orthologs in promoting terminal, neuronogenic progenitors**. **(A) **In *C. elegans*, EGL-46 (green) is expressed in late neuronal progenitors destined to terminally divide to produce two neurons (N/N divisions; some of these daughters undergo programmed cell death and are labeled X) and transiently in the nascent neurons. EGL-46 is not expressed in early neural progenitors, which divide to generate more progenitors (P/P or P/N divisions). **(B) **In *egl-46 *mutants, cells that would normally terminally divide instead undergo proliferative divisions, thus delaying terminal, neuronogenic divisions. **(C) **In the embryonic mammalian OE early progenitor cells divide apically (P/P) to produce more progenitors and/or sustentacular cells (which do not display markers of differentiation until later stages). Some of the apically produced cells migrate to the basal lamina, where they divide again to produce neurons (some of these basal progenitors undergo N/N divisions). By E14.5 apical and neuro-basal cells are distinguished based on their nuclear morphology, which is elongate in the apical layer. By E16.5, apical progenitors are believed to produce sustentacular cells, basal progenitors are believed to produce developing neurons, and these populations are believed to be increasingly separate, with diminishing migration from the apical to the basal population. Cells known to express *Insm1 *are indicated in purple. **(D) ***Insm1*^*-/- *^OEs contain more apical and presumed proliferative progenitors and fewer basal, terminally dividing and presumed neuronogenic progenitors, a phenotype similar to that caused by *egl-46 *mutation in nematodes (compare (B) and (D)). This leads to a thicker apical layer (delineated by a dashed line in (C, D)) and a thinner neuro-basal layer. In contrast to *egl-46 *mutant nematodes, *Insm1*^*-/- *^mouse embryonic OEs display fewer neurons, and at a later stage increased apoptosis. M represents cells in mitosis, N neurons, Su sustentacular cells and QL a neuroblast of the nematode lineage.

Previous studies in the embryonic OE have shown that *Insm1 *is expressed in basal progenitors during mitosis and in intermediate cells, some of which are undergoing mitosis but many others are no longer dividing (Ki67-negative; shown at E14.5 in Figure 9 of [15]). Furthermore, *Insm1 *was not expressed in most apical progenitors or in many TuJ1-expressing neurons [15,16]. By combined *in situ *hybridization and IHC we confirmed that, in the embryonic OE, *Insm1 *mRNA is expressed in intermediate and basal cells, including all progenitors undergoing mitosis basally (all 29 basal mitoses from 6 sections of OE at E12.5 and all 19 basal mitoses from 2 sections at E14.5 expressed *Insm1*; Figure 9 and similar data not shown), and most of the progenitors undergoing mitosis at intermediate positions (defined as more than 2 nuclei from the base or the apex; 11 intermediate mitoses out of 14 at E12.5 and all 7 intermediate mitoses at E14.5 expressed *Insm1*; Figure 9 and similar data not shown). By contrast, at the same stages *Insm1 *mRNA is rarely present in apical mitoses, although it is in some apical cells (at E12.5, only 1 of 12 apical cells expressing *Insm1 *was in mitosis, whereas 48 of 49 apical mitoses did not express *Insm1*; at E14.5, only 1 of 13 apical cells expressing *Insm1 *was in mitosis, whereas 21 of 22 apical mitoses did not express *Insm1*; Figure 9 and similar data not shown). Together, the data suggest that *Insm1 *is activated in apical cells transitioning towards the base, stays on during basal divisions and is turned off in postmitotic cells as they begin to differentiate into neurons (Figure 10C). This pattern of expression is reminiscent of that of *egl-46 *in nematode neuronal lineages (Figure 10A). One potential difference is that in nematodes *egl-46 *is expressed exclusively in terminally dividing (N/N) progenitors, whereas *Insm1 *seems to be expressed in these as well as in the earlier, presumably proliferative progenitors transitioning from apex to base. This presumed onset of expression in transitioning progenitors is consistent with the defect in apical to basal progenitor transitions we have observed in *Insm1*^*-/- *^OEs.

## Discussion

*Insm1 *encodes a zinc-finger protein that is expressed in late progenitors and nascent neurons throughout the developing nervous system, including the embryonic OE [15,16]. Here we show several lines of evidence indicating that, in embryonic OE, deletion of *Insm1 *causes a decrease in terminal, neuronogenic progenitors and an increase in earlier, proliferative progenitors. First, at certain embryonic stages the OEs of *Insm1*^*-/- *^mice (compared with OEs of *Insm1*^*+/+ *^mice) contain more apical mitoses (E12.5) and fewer basal ones (E12.5 and E14.5). At these stages, progenitors undergoing mitosis apically generate more progenitors, whereas some progenitors undergoing mitosis at the basal lamina generate neurons. Second, at E12.5 the OEs of *Insm1*^*-/- *^mice display more cells expressing ASCL1 (a marker of TAPs, which migrate from apical to basal locations but still divide to produce progenitors) and fewer cells expressing NEUROD1 (a marker of INPs, which divide terminally next to the basal lamina to produce neurons). Third, by sequential incorporation of nucleoside analogs we demonstrate that, at E12.5, the OEs of *Insm1*^*-/- *^mice contain fewer progenitors that are terminally dividing. These results indicate that *Insm1 *promotes the transition of olfactory progenitors from apical, proliferative and uncommitted to basal, terminally dividing and neuron-producing.

### Potential molecular mechanisms of Insm1 action

This study does not address the molecular mechanism by which Insm1 regulates the progression from early to late neuronal progenitors. In this regard, studies on EGL-46 offer no clues as its mechanism of action also has not been explored. Some insight, however, might be drawn from previous work revealing that Insm1 has at least two molecular mechanisms of action: it can bind promoter elements to repress transcription of certain genes and it can also promote cell cycle arrest and exit [5]. The basic helix-loop-helix transcription factor ASCL1 binds to an element in the promoter of *Insm1*, and *Ascl1 *is required for *Insm1 *transcriptional activation in the sympathoadrenal lineage [47], ganglionic eminences [48] and OE [16]. Furthermore, in the sympathoadrenal lineage, ganglionic eminences and cortex, ablation of *Insm1 *led to an up-regulation of *Ascl1*, and these results were interpreted as evidence that Insm1 represses *Ascl1 *[16,23]. Similarly, the increase in ASCL1-expressing cells in the OE of *Insm1*^*-/- *^embryos here reported could result from direct transcriptional repression. However, this increase in ASCL1-expressing progenitors does not explain the decrease in basal progenitors and neurons, both of which require ASCL1.

Insm1 also binds an element in the *NeuroD1 *promoter and represses its transcription in pancreatic islet cell development [5,46]. However, this regulatory interaction fails to explain the decrease of NEUROD1-expressing cells in the absence of Insm1. Alternatively, the retention of OE progenitors in a proliferative state in the absence of Insm1 might be more in keeping with the proposed role of this zinc-finger protein in cell cycle exit [5,49].

### Anatomical consequence of *Insm1 *ablation: increase in apical and decrease in basal cells and neurons

Our results do explain the anatomical changes observed in Insm1^-/- ^OEs. The increase in apical progenitors observed at E12.5 may account for the increase in cells observed in the apical layer at subsequent stages (E14.5 and E18.5). Many of these are likely sustentacular cells or their precursors because by E14.5 there is no longer an increase in apical progenitors. Likewise, the decrease of progenitors that are basal, NEUROD1-positive and terminally dividing at E12.5 may explain the decrease in nascent and mature neurons observed at this and later stages. Although apoptosis may also contribute to the decrease in neurons, its later onset (E14.5) and infrequent occurrence (100 times fewer cells appear undergoing programmed cell death than neurons are missing) indicates that it cannot be the sole or principal cause for neuronal decrease. The net increase in apical progenitors and cells and net decrease in basal progenitors and neurons seem to compensate for each other during the stages examined here because we never detected an overall difference in the size (measured in thickness, cell density or surface area in Figure 2 and Additional files 4 and 5) between *Insm1*^*-/- *^and *Insm1*^*+/+ *^OEs.

### Potential roles of *Insm1 *in cell survival and neuronal differentiation

The slight increase in cell death detected in *Insm1*^*-/- *^OEs might also be suggestive of a role of Insm1 in cell survival. This role would be modest, since it affects a very small fraction of *Insm1*^*-/- *^OE cells during the period examined (from E10.5 to E18.5). However, the increase in cell death is observed at a stage (E14.5) when olfactory receptor neurons innervate their targets in the olfactory bulb. In the wild type, the peak of cell death at this moment is thought to reflect the elimination of neurons that failed to establish proper synaptic contacts [32]. Hence, given that the *Insm1*^*-/- *^embryos may also experience a reduction in olfactory bulb neurons (as they do in cortical neurons [16]), the slight increase in OE cell death may be an indirect consequence and not reflect a cell autonomous role of Insm1 in promoting neuronal survival.

The decrease in OMP-expressing, mature olfactory receptor neurons is more pronounced than the decrease in TuJ1-expressing, immature neurons (approximately 40% versus approximately 92%, respectively; Figure 3). The increase in cell death mentioned above could contribute to this difference if the cells dying are neurons prior to maturation. We were not able to elucidate which cells undergo programmed cell death in the OE, but previous studies have shown these to be TuJ1-expressing, young neurons [32]. Although the increase of dying (that is, ACC3-expressing) cells (0.3 cells per 0.1 mm^2 ^at E14.5) is ten-fold lower than the decrease in OMP-expressing neurons (3.1 cells per 0.1 mm^2 ^at E14.5; after correcting for the 39% decrease in immature, TuJ1-expressing neurons), the transient nature of apoptotic cells underestimates their frequency. For example, if an apoptotic cell expresses ACC3 during a 3-hour period (a guess, since this has not been measured), then the density of missing cells (dying at a density of 0.3 cells per 0.1 mm^2 ^mentioned above) would reach 3 per 0.1 mm^2 ^in 30 hours, a value consistent with the observed reduction in OMP-expressing neurons.

The disproportionately larger reduction in OMP-expressing mature neurons than in TuJ1-expressing young neurons might also suggest a role for Insm1 in neuronal differentiation. In fact, Insm1 has been implicated in the differentiation of pancreatic and intestinal endocrine cells [19], adrenal chromaffin cells [23] and hindbrain monoaminergic neurons [24]. However, the disproportionate reduction in OMP-expressing cells may simply reflect a more steep change in the onset of OMP expression than in the onset of TuJ1 expression during the observed stages (E14.5 and E18.5). Due to the lethality of *Insm1*^*-/- *^embryos, we cannot determine whether all TuJ1-expressing nascent neurons will mature to express OMP, or whether many of them will fail to differentiate (thus revealing a role for Insm1 in olfactory neuron differentiation).

### Phenotypic similarities between *Insm1 *and *egl-46 *mutants suggest functional conservation in neurogenic proliferation from nematodes to mammals

Mammalian *Insm1 *and nematode *egl-46 *have a similar pattern of expression in neuronal lineages: both are transiently expressed in neuronogenic progenitors and nascent neurons and not in many of the earlier, proliferative progenitors (Figure 10A, C). In particular, nematode *egl-46 *is expressed in progenitors of the motorneuron and Q-neuroblast lineages undergoing N/N but not P/P or P/N divisions, whereas mouse *Insm1 *is expressed in OE progenitors undergoing mitosis basally (many of which are neuronogenic) but not apically (many of which are proliferative). Although our data cannot determine whether *Insm1 *is exclusively expressed in terminally dividing N/N progenitors of OE, they indicate that *Insm1 *is expressed in late neuronogenic progenitors and not in many of the earlier, proliferative progenitors. In nematodes, mutation of *egl-46 *results in a transient decrease in neuronogenic (N/N) divisions and a concomitant increase in proliferative (P/N or P/P) divisions (Figure 10B). This occurs because progenitors that by lineage are expected to terminally divide fail to do so, and instead some of their progeny divide one or more times. Similarly we find that, in the mammalian OE, deletion of *Insm1 *results in fewer terminally dividing neuronogenic progenitors and in a concomitant increase in proliferative (apical and ASCL1-expressing) progenitors (Figure 10D). Hence, we propose that *Insm1 *and *egl-46 *play evolutionarily conserved roles in promoting terminal, neuronogenic divisions.

It should be noted that, in both organisms, the mutant effects are modest. In *egl-46 *mutants, only some terminal progenitors are delayed, and only by one or two cell divisions [2-4]. In *Insm1 *mutants, the increase in apical progenitors is transient (detected at E12.5 but not at E10.5 or E14.5) and small (42% increase; Figure 6), as is the decrease in basal progenitors (45% at E12.5 and 35% at E14.5; Figure 6). The subtlety of both phenotypes demonstrates that these two genes are not essential for the transition from proliferative to neuronogenic progenitors, and instead suggests that their function is to fine tune their occurrence.

A difference between the *egl-46(loss-of-function) *and *Insm1*^*-/- *^phenotypes is that, in mutant nematodes, an increase in proliferative divisions eventually results in additional neurons, whereas mouse *Insm1*^*-/- *^OEs still contain fewer neurons than the wild type at the latest stages examined (E18.5). The embryonic lethality of *Insm1*^*-/- *^prevents determining whether more neurons would be eventually produced. However, in these mutant OEs we also detected an increase in apical cells, which would eventually become sustentacular glia. Because apical progenitors stop transitioning basally by E16.5 [1,25], the increase in apical progenitors observed at E12.5 may eventually result in more sustentacular cells and not in more neurons.

### Phenotypic and other similarities suggest a common mechanism of neurogenic proliferation between OE and cortex

In embryonic cortex, progenitors undergoing mitosis at the apical side of the ventricular zone generate more progenitors, some of which migrate in the basal direction. These basally located progenitors divide one or more times to produce neurons, which migrate to the cortical plate and differentiate [50-55]. The effects of *Insm1 *deletion in OE reported here share similarities with those reported in dorsal telencephalon [16]. In both embryonic OE and cortex, *Insm1 *deletion results in an enlargement of the area with apical progenitors (the ventricular zone in cortex and the apical layer in OE), a decrease in basal, neuronogenic progenitors (the intermediate progenitor cells of the cortical subventricular zone and the INPs of OE), and a decrease in neurons. Furthermore, both neuroepithelia share striking similarities that extend beyond the *Insm1*^*-/- *^phenotype. At embryonic stages, both have progenitors dividing apically (near the airways in OE and near the ventricle in cortex), some of which display radial glia characteristics [56]. These apical progenitors undergo interkinetic nuclear migration and generate glial-like epithelial cells (ependymal in cortex and sustentacular in OE) as well as additional progenitors that migrate basally (to the basal lamina in OE and to the subventricular zone in cortex). In both embryonic neuroepithelia, many of these basal progenitors divide terminally to produce neurons. After embryogenesis, proliferation subsides in apical areas but is maintained in basal areas (subventricular zone and base of OE), where adult neural stem cells reside and neurogenesis occurs. All these similarities suggest that both neuroepithelia share a common mechanism of progenitor migration, proliferation and differentiation orchestrating neurogenesis and gliogenesis. We propose that common molecular pathways involving Insm1 and other factors may direct the development of OE and cortex and, perhaps, other neural tube and placodal neuroepithelia.

## Conclusions

Our results indicate that, in the mouse embryonic OE, *Insm1 *promotes the transition of progenitors from apical, proliferative and uncommitted to basal, terminally dividing and neuron-producing. This role is similar to that of *Insm1*'s *C. elgans *ortholog, *egl-46*, in certain neuronal lineages, and therefore appears to be evolutionarily conserved (Figure 10). Furthermore, the role of *Insm1 *in embryonic OE is similar to its role in embryonic cortex, in both cases facilitating the progenitors' transition from apical, proliferative and uncommitted to basal, terminally dividing and neuron-producing (Figure 10). Based on this and other similarities, we propose that both the neural-tube derived cortex and the placode-derived OE contain homologous neuroepithelial organization and types of progenitors whose progression is governed by common factors such as Insm1.

## Materials and methods

### Generation of *Insm1 *knockout mouse

Construction of *Insm1 *targeting vector. A mouse 129/SvEv genomic BAC library, RPCI-22, was screened using a mouse *Insm1 *3' UTR probe isolated from pCRII7 [15] by digesting with *Eco*RI. Three BAC clones were isolated and mapped using restriction enzymes. Two subclones from BAC clone 439G2 were used to generate the *Insm1 *targeting vector (Figure 1A). A 15.2 kb *Bgl*II genomic fragment containing the *Insm1 *coding sequence, 6.9 kb of 5' sequence and 6.7 kb of 3' sequence was cloned into the *Bgl*II site of pCMV-Myc (Clontech, Mountain View, California, USA) to generate pCMV-Myc G2Bgl. A 6-kb *Eco*RI fragment from the G2 BAC was cloned into the *Eco*RI site of pBluescript-SK- (Stratagene, Lost Pines, Texas, USA) to generate pSK-G2Eco24. A 1.05-kb *Xba*I-*Eco*RI genomic fragment from pSK-G2Eco24, the 3' homology arm, was cloned into pSK-, which had been cut with *Sm*aI and *Eco*RI, to generate pSK-3'arm. The thymidine kinase cassette of pSK-PGKTK (a gift of Dr L Jameson) was subcloned as an *Eco*RI-*Xho*I fragment into pSK-3'arm to generate pSK-3'armTK. The floxed neomycin (Neo) cassette was released from pSK-FloxPGKNeo (a gift of Dr L Jameson) by cutting with *Xba*I; after blunting, the floxed Neo cassette was subcloned in an inverted orientation into pSK3'armTK that had been cut with *Bam*HI and blunted to create pSKneo3'armTK. A 4-kb 5' fragment, the 5' homology arm, was cut from pCMV-Myc G2Bgl and subcloned into pSKneo3'armTK, which had been cut with *Xba*I and blunted, to generate the targeting vector, pSK insm1KO#2.

Generation of mouse ES cells and chimeric mice containing a targeted deletion of *Insm1 *The targeting vector pSKinsm1KO#2 was linearized with *Not*I prior to electroporation. Electroporation, ES cell culture and blastocyst injections were performed by the Northwestern University Transgenic and Targeted Mutagenesis Laboratory. Homologous recombination in positive ES cell clones was confirmed by PCR with external primer insm1 A7(CGAACATCCATACATTGCCCTGTAGCTCAG) and primer neoA1(CTGCTAAAGCGCATGCTCCAGACTGCCTTG), which yield a 1.3-kb band from the recombined allele, and by Southern hybridization of *Bgl*II digested genomic DNA with a 168-bp 5' probe, which detects a 15-kb wild-type band and a 6.35-kb recombined band, and a 3' probe that detects a 15-kb wild-type band and a 7.5-kb recombined band.

#### Germline transmission

Chimeric males were mated to C57Bl/6 (Taconic) females, and germline transmission was indicated by agouti offspring. PCR with primers insm1 A7 and neoA1 demonstrated the presence of the targeted allele. The resulting mixed background 129P/Ola+C57Bl/6 *Insm1*^- ^*Neo*^*+ *^line (*Insm1*^*tm1Jga*^) was crossed against a C57Bl/6 E2a-Cre trasnsgenic line in order to excise the Neo cassette. The resultant *Insm1*^- ^transline (*Insm1*^*tm1.1Jga*^) was then backcrossed into C57Bl/6 mice (strain code 027, Charles River, Wilmington, MA, USA) for ten generations to achieve effective line purity. Additionally, mice from the mixed-background *Insm1*^- ^line were out-crossed into the CD-1 strain (strain code 002, Charles River) and back-crossed for ten generations.

Mouse genotypes were ascertained by tail samples purified by DNEasy DNA purification kit (69506, Qiagen, Gaithersburg, MD, USA), followed by PCR-based genotyping for the KO allele. PCR for the KO allele used the forward primer 5'-TTTGGAAGCCAGCCAGCA-3' and the reverse primer 5'-CCCGATTCTTGTGAGAGAGGAGGA-3'. PCR for the wild-type allele used the same forward primer, but used reverse primer 5'-GGGAACGCGCAACTCAACTC-3'. Primers were synthesized by Integrated DNA Technologies (Coralville, IA, USA). Due to the GC-rich nucleoside content of the *Insm1 *sequence, 1M betaine (B2629, Sigma-Aldrich, St Louis, MO, USA) was added to the reaction mixture to promote DNA melting.

### Generation of homozygous knockout embryos

Heterozygous mice were cross-bred in timed pregnancies in order to produce embryos of specific, known ages. Males and females were placed together for a single night and the following morning were checked for plugs and separated whether a plug was observed or not. Midnight of the night of mating was considered the beginning of E0. In order to rescue prenatal lethality of homozygous KO embryos collected at E12.5 and later, pregnant dams were supplied daily with L-DOPA (1 mg/mL, 0.25% ascorbic acid) starting no later than 7 dpc (TCD0600, VWR Scientific, West Chester, PA, USA; D9628, Sigma-Aldrich) [23,26]. To obtain embryos at E11.5 and earlier, females in which plugs had been observed were collected for harvest. In order to collect embryos of developmental stages E12.5 and later, females were weighed at least once prior to 10.5 dpc, then every day at 10.5 dpc and later in order to track weight gain. Females in which consistent weight gain of at least 5 to 10% per day was observed were considered pregnant and were collected for harvest.

Dams were sacrificed by isoflurane overdose, followed by cervical dislocation. Embryos were harvested, and their tails were removed, washed in PBS, and processed for genotyping by PCR. KO embryos and either wild type (WT) or heterozygous (HET) littermates were assayed for developmental stage [57], decapitated, and placed immediately into 4% paraformaldehyde fixative (1008A, Tousimis, Rockville, MD, USA). Following dehydration in sucrose gradient, embryos were weighed, and the heads were embedded in OCT compound (25608-930, VWR Scientific) and cryosectioned.

### *In situ *hybridizatioin, immunohistochemistry, nucleoside analog labeling, and EdU detection chemistry

*In situ *hybridization and IHC were performed on 10-μm or 12-μm sections as previously described [15]. For IHC, the following primary antibodies were used: mouse anti-neuronal class III β-tubulin (1:500; clone Tuj1, Covance, Princeton, NJ, USA), goat anti-OMP (1:1,000; Wako Chemicals, Richmond, VA, USA), rabbit anti-pH3 (1:100; Millipore, Billerica, MA, USA), rabbit anti-ACC3 (1:1,000; Cell Signaling, Beverly, MA, USA), rat anti-BrdU (1:400; AbD Serotec, Raleigh, NC, USA), mouse anti-BrdU clone MoBu (1:50; Santa Cruz Biotechnology, Inc., Santa Cruz, CA, USA), mouse anti-Ki67 (1:200; BD Pharmingen, San Diego, CA, USA), mouse anti-Mash1 (1:100; BD Pharmingen), goat anti-NEUROD1 (1:50; Santa Cruz Biotechnology, Inc.).

Anti-Tuj1, anti-Ki67, and anti-ASCL1 staining required antigen retrieval with 10 mM sodium citrate in 0.25% Triton X-100 at 96°C, pH6.0 for 20 minutes, with a 30-minute cool-down, prior to blocking. Anti-BrdU staining required antigen exposure in 5 M HCl in 1% Triton X-100 for 15 minutes, prior to blocking. DAPI counterstain was applied to all IHC experiments, with the exception of those requiring the detection of BrdU because the acid antigen exposure ablates DAPI staining. Staining for ACC3 was performed using a horseradish peroxidase conjugated secondary antibody and a colorimetric 3,3'-diaminobenzadine (DAB) reaction kit (SK-4100, Vector Labs, Burlingame, CA, USA) to visualize positively stained cells. Detection followed the supplied protocol.

For BrdU/Ki67 double labeling, and for analysis of S-phase alone (BrdU only) 10 mM BrdU (B23151, Invitrogen, Carlsbad, CA, USA) was injected into pregnant dams (50 mg/kg) 30 minutes prior to sacrifice. For EdU/BrdU timed pulse labeling, 10 mM EdU (A10044, Invitrogen) was injected at 12.5 dpc (25 mg/kg), followed 12 hours later (13 dpc) by 10 mM BrdU (30 mg/kg). Embryos were allowed to develop until E14.5, at which point they were harvested and cryosectioned as described above. The mouse primary antibody to BrdU (clone MoBu) was used for IHC detection of BrdU because of demonstrated lack of cross-reactivity with EdU. Following IHC detection of BrdU, EdU was detected according to a modified protocol supplied by Invitrogen. Briefly, following BrdU IHC, slides were washed in 1× PBS, incubated in the EdU reaction cocktail (for 1.5 ml of reaction cocktail: 7.5 μl component B (AlexaFluor 488 azide), 30 μl component H (copper sulfate), 1,313 μl component G (reaction buffer), 150 μl component I (buffer additive), washed in 1% bovine serum albumin in 1× PBS, then cover-slipped.

### Quantification and statistics

Littermates were paired, and stained sections were visualized and photographed on a Nikon Eclipse E600 microscope with an RT Slider SPOT camera (version 2.3.1; Diagnostic Instruments, Sterling Heights, MI, USA). Coronal sections were aligned anatomically to assure that similar regions of OE were being compared between KO embryos and their littermates. Images were counted blindly using Image J software (available at [58]) with the Cell Counter plugin (available at [59]). BrdU/EdU co-labeling and BrdU/Ki67 co-labeling were evaluated using the Colocalization plugin (available at [60]). Images were optimized for brightness and contrast using Image J and CorelDraw software. Data were cataloged and analyzed using Microsoft Excel 2007. For measurements of the thickness of the OE, anatomically aligned section pairs were measured multiple times (seven for E12.5, eight for E14.5, and nine for E18.5) along the straight portion of the septal OE. These measurements were summed to generate an average OE thickness. These paired averages were then compared to generate a percent difference in thickness between the KO and wild-type littermates. All other measurements were obtained from all areas of OE. A one-sample Student's *t*-test was used to evaluate the percent difference between the measurements of experimental (KO) and control (wild-type or HET) groups for both cell counts and for thickness measurements. Due to comparatively infrequent expression of ACC3, sections were not anatomically aligned, and all of the sections from one or more whole slides were quantified for positive staining of ACC3. Because the sections were not aligned anatomically for ACC3 expression, an unpaired Student's *t*-test was used for statistical analysis. To compare the mean weights of KO embryos to wild-type+HET embryos, an unpaired *t*-test was used. For all graphs, error bars reflect standard error of the mean (SEM) and *P ≤ 0.05, **P ≤ 0.01, ***P ≤ 0.001.

## Abbreviations

ACC3: activated cleaved caspase 3; BAC: bacterial artificial chromosome; bp: base pair; BrdU: 5-bromo-2-deoxyuridine; DAPI: 4',6-diamidino-2-phenylindole; dpc: days post-coitus; E: embryonic day; EdU: 5-ethynyl-2'-deoxyuridine; ES: embryonic stem; IHC: immunohistochemistry; KO: knockout; INP: immediate neuronal precursor; OE: olfactory epithelium; OMP: olfactory marker protein; PBS: phosphate-buffered saline; pH3: phosphohistone H3; TAP: transit amplifying progenitor; UTR: untranslated region.

## Competing interests

AD and JG-A are married. The authors declare that they have no competing interests.

## Authors' contributions

JNR contributed to characterizing the *Insm1 *KO phenotype by performing most mouse husbandry, IHC, photography, quantification, plotting and statistical analysis of results, made comments on the manuscript and wrote portions of it (figures, legends and Materials and methods). AD initiated the project, contributed to determining the expression pattern of *Insm1*, formulated the working hypotheses regarding the role of *Insm1 *in neurogenic progenitors, designed and generated the *Insm1 *KO mice, initially detected their olfactory phenotype, noticed similarities between OE and cortex, made comments on the manuscript and wrote portions of it (Materials and methods). JG-A assisted in the determination of the expression pattern of *Insm1 *and in the generation of *Insm*1 KO mice, planned and supervised their phenotypic characterization, analyzed and interpreted results, noticed the similarities between *egl-46 *and *Insm1 *and between OE and cortex, and wrote most of the manuscript (title, abstract, Background, Results, Discussion, references, figures and legends).

## Supplementary Material

Additional file 1 **Deletion of *Insm1 *does not affect the weight of embryos throughout development**. **(A) **When *Insm1*^*-/- *^embryos and their *Insm1*^*+/+ *^and *Insm1*^*+/- *^littermates are compared by weight, no statistically significant difference can be detected at any developmental time point. **(B) **When weights are normalized to the combined *Insm1*^*+/+ *^plus *Insm1*^*+/- *^weights at every stage, there is also no detectable statistically significant difference with the weights of *Insm1*^*-/- *^embryos. Panel (A) is plotted logarithmically on the y-axis in order to allow easier comparison of different stages to one another. Data are presented as mean values ± standard error of the mean (SEM). Click here for file

Additional file 2 **Deletion of *Insm1 *does not significantly alter markers of embryonic stage**. Selected Theiler staging criteria were compared between knockout embryos and their littermates at every stage collected. No consistent difference could be detected between the *Insm1*^*-/- *^embryos and *Insm1*^*+/+ *^or *Insm1*^*+/- *^embryos. Topmost boxes indicate the embryonic stage (Theiler stage number in parentheses) that includes the criteria listed beneath it. Leftmost boxes indicate the day at which the litter was collected. Percentages of embryos determined to have the characteristic out of embryos examined are highlighted in yellow. Grayed out boxes indicate characteristics not assessed at the given time point. Click here for file

Additional file 3 ***Insm1*^*-/- *^embryos exhibit a prenatal lethality that might be partially rescued by administration of L-DOPA**. *Insm1*^*-/- *^embryos are retrieved at lower than expected Mendelian frequencies beginning at E12.5. Prenatal administration of L-DOPA may have facilitated the recovery of embryos at later stages (E17.5 and later). Click here for file

Additional file 4 **Apical layer enlargement and neuro-basal layer reduction in the OE of *Insm1*^*-/- *^mice**. **(A) **At E12.5, no difference in the thickness of the septal OE is detected between *Insm1*^*-/- *^mice and their *Insm1*^*+/+ *^littermates (*P *= 0.98; n = 3 embryo pairs, total of 9 aligned section pairs). By E14.5, the apical layer of the OE is clearly distinguishable from the rest of the OE based on elongate nuclear morphology and increased cell density. At E14.5, despite no change to the overall thickness of the OE in *Insm1*^*-/- *^mice, the average thickness of the apical layer is 47% greater and the remaining neuro-basal layer is 20% thinner (*P *= 0.84 overall; *P *< 0.001 apical; *P *< 0.01 neuro-basal; n = 5 embryo pairs, total of 13 aligned section pairs). By E18.5, the average thickness of the apical layer in the mutants is 59% greater and the remaining neuro-basal layer is 26% thinner, but only a slight reduction (8.7%) in the overall thickness of the OE is detected (*P *= 0.0502 overall; *P *< 0.05 apical; *P *< 0.01 neuro-basal; n = 3 embryo pairs, total of 9 aligned section pairs). Data are presented as mean values ± standard error of the mean (SEM). Click here for file

Additional file 5 **Size of OE does not differ between *Insm1*^*-/- *^(KO) and *Insm1*^*+/+ *^(WT) embryos**. Areas of OE were measured from paired coronal sections of littermate embryos. Each value was obtained from the average of three to six sections per embryo, with n = 3 embryos. Click here for file
