# Supplementary figures and images for: Insm1 promotes the transition of olfactory progenitors from apical and proliferative to basal, terminally dividing and neuronogenic
Source: Neural Dev. 2011 Feb 1;6:6. doi: 10.1186/1749-8104-6-6 (PMC3057173; doi:10.1186/1749-8104-6-6)

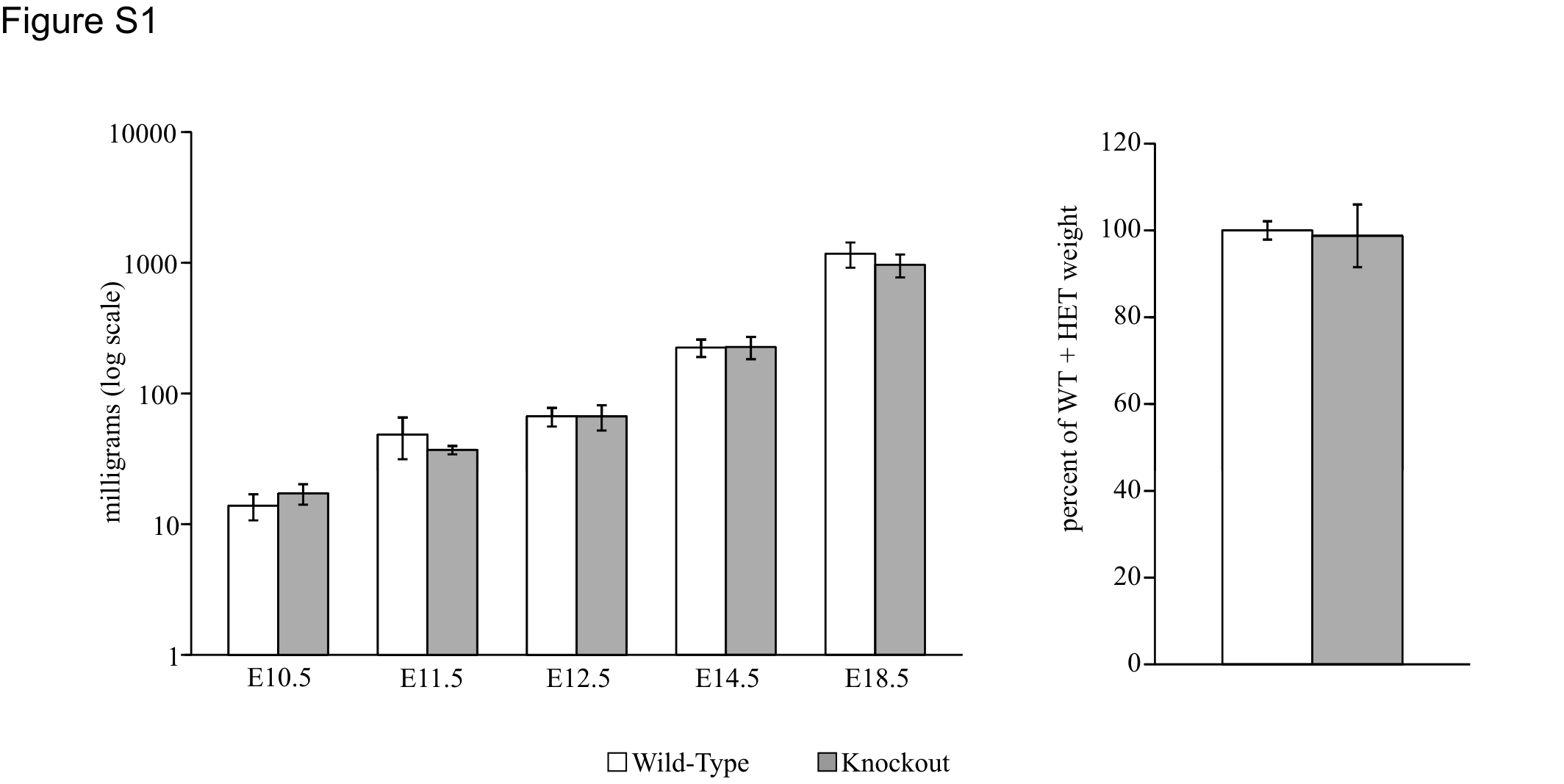

Supplement: Additional file 1 — Deletion of Insm1 does not affect the weight of embryos throughout development. (A) When Insm1-/- embryos and their Insm1+/+ and Insm1+/- littermates are compared by weight, no statistically significant difference can be detected at any developmental time point. (B) When weights are normalized to the combined Insm1+/+ plus Insm1+/- weights at every stage, there is also no detectable statistically significant difference with the weights of Insm1-/- embryos. Panel (A) is plotted logarithmically on the y-axis in order to allow easier comparison of different stages to one another. Data are presented as mean values ± standard error of the mean (SEM). [file 1749-8104-6-6-S1.TIFF]

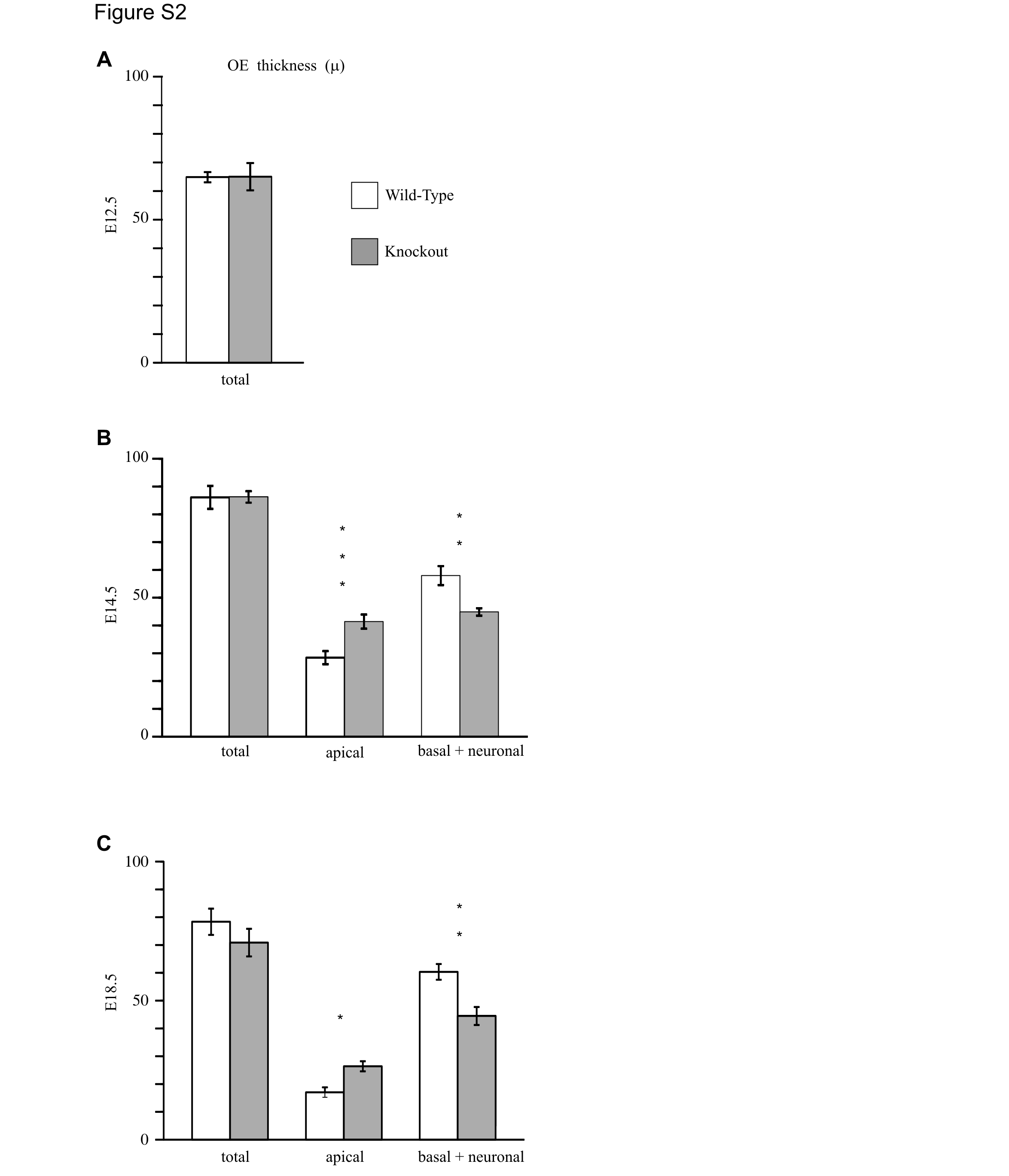

Supplement: Additional file 4 — Apical layer enlargement and neuro-basal layer reduction in the OE of Insm1-/- mice. (A) At E12.5, no difference in the thickness of the septal OE is detected between Insm1-/- mice and their Insm1+/+ littermates (P = 0.98; n = 3 embryo pairs, total of 9 aligned section pairs). By E14.5, the apical layer of the OE is clearly distinguishable from the rest of the OE based on elongate nuclear morphology and increased cell density. At E14.5, despite no change to the overall thickness of the OE in Insm1-/- mice, the average thickness of the apical layer is 47% greater and the remaining neuro-basal layer is 20% thinner (P = 0.84 overall; P < 0.001 apical; P < 0.01 neuro-basal; n = 5 embryo pairs, total of 13 aligned section pairs). By E18.5, the average thickness of the apical layer in the mutants is 59% greater and the remaining neuro-basal layer is 26% thinner, but only a slight reduction (8.7%) in the overall thickness of the OE is detected (P = 0.0502 overall; P < 0.05 apical; P < 0.01 neuro-basal; n = 3 embryo pairs, total of 9 aligned section pairs). Data are presented as mean values ± standard error of the mean (SEM). [file 1749-8104-6-6-S4.PDF]
