# Supplementary material for: Insm1 promotes the transition of olfactory progenitors from apical and proliferative to basal, terminally dividing and neuronogenic
Source: Neural Dev. 2011 Feb 1;6:6. doi: 10.1186/1749-8104-6-6 (PMC3057173; doi:10.1186/1749-8104-6-6)
Supplement: Additional file 3 — Insm1-/- embryos exhibit a prenatal lethality that might be partially rescued by administration of L-DOPA. Insm1-/- embryos are retrieved at lower than expected Mendelian frequencies beginning at E12.5. Prenatal administration of L-DOPA may have facilitated the recovery of embryos at later stages (E17.5 and later). [file 1749-8104-6-6-S3.PDF]

		W/O L-DOPA			
		WT	*HET*	*KO*	***% KO***
**dpc**	*11.5*	6	16	7	**24.14**
	12.5	11	18	7	**19.44**
	13.5	27	50	11	**12.50**
	14.5	24	41	13	**16.67**
	15.5	19	42	16	**20.78**
	16.5	15	35	1	**1.96**
	17.5	3	21	2	**7.69**
	18.5	10	18	0	**0.00**
	20.5	2	4	0	**0.00**

		W/ L-DOPA			
		WT	*HET*	*KO*	***% KO***
**dpc**	*11.5*	2	10	3	**20.00**
	12.5	9	33	7	**14.29**
	13.5	0	0	0	**N/A**
	14.5	39	85	12	**8.82**
	15.5	0	0	0	**N/A**
	16.5	0	2	0	**0.00**
	17.5	3	9	2	**14.29**
	18.5	29	56	5	**5.56**
	20.5	4	11	2	**11.76**
