# Supplementary material for: Insm1 promotes the transition of olfactory progenitors from apical and proliferative to basal, terminally dividing and neuronogenic
Source: Neural Dev. 2011 Feb 1;6:6. doi: 10.1186/1749-8104-6-6 (PMC3057173; doi:10.1186/1749-8104-6-6)
Supplement: Additional file 5 — Size of OE does not differ between Insm1-/- (KO) and Insm1+/+ (WT) embryos. Areas of OE were measured from paired coronal sections of littermate embryos. Each value was obtained from the average of three to six sections per embryo, with n = 3 embryos. [file 1749-8104-6-6-S5.DOC]

| Stage | WT OE section size average ± SEM (µm^2^) | KO OE section size average ± SEM (µm^2^) | Student’s t-test |
| --- | --- | --- | --- |
| E10.5 | 26,757 ± 4,022 | 22,854 ± 3,930 | p = 0.33 |
| E11.5 | 44,615 ± 5,405 | 38,790 ± 7,406 | p = 0.26 |
| E12.5 | 78,596 ± 7,926 | 77,053 ± 2,293 | p = 0.81 |
| E14.5 | 105,848 ± 4901 | 106,639 ± 5,229 | p = 0.93 |
| E18.5 | 90,005 ± 11,762 | 78,102 ± 5,181 | p = 0.38 |

**Table S3. Size of OE does not differ between *Insm1^-/-^*****(KO) and** ***Insm1^+/+^*** **(WT) embryos.** Areas of OE were measured from paired coronal sections of littermate embryos. Each value was obtained from the average of 3-6 sections per embryo, with n = 3 embryos.
